# Life and love under criminalization: The experiences of people living with HIV in Canada

**DOI:** 10.1371/journal.pone.0306894

**Published:** 2024-07-25

**Authors:** Christopher Tatham

**Affiliations:** Department of Sociology and Anthropology, University of Guelph, Guelph, Ontario, Canada; University of Technology Sydney, AUSTRALIA

## Abstract

Based upon qualitative interviews with 54 women and men living with HIV across Ontario, Canada, this paper examines the impact of HIV criminalization on the sexual and romantic relationships of people living with HIV. This research highlights the navigation strategies people living with HIV create and employ to both navigate and protect themselves from the law. Through a thematic and intersectional analysis, this study shows how adoption of these strategies is unequal, with access to navigation strategies varying along lines of gender, race, and sexual orientation. As a result, women and racialized people living with HIV face more difficulties navigating the impact of the law. HIV criminalization in Canada fuels and validates HIV stigma and produces vulnerability both within and outside of the relationships of people living with HIV. This paper seeks to understand HIV criminalization from the perspective of those governed by the law, in hopes of producing knowledge which will contribute to legal reform, inform policy, and support the development of efficacious secondary prevention initiatives.

## Introduction

This study aims to provide insight into the ways that HIV criminalization legislation in Canada impacts the everyday lives of people living with HIV (PLWH), particularly in terms of their intimate relationships. Through qualitative interviews with 54 PLWH, this paper extends understandings of HIV criminalization and HIV disclosure by documenting the strategies PLWH create to protect themselves from prosecution and navigate the disruptive impact the law has within their lives. Through drawing on intersectionality, this work illustrates how access to these navigation strategies is patterned along lines of gender, race, and sexual orientation. In so doing, this paper argues that women and racialized PLWH face increased and differently nuanced hardship from the law, in comparison to gay and straight men living with HIV.

This research illustrates the broad harmful effects of HIV criminalization from the perspectives of those most directly impacted by legislation and underscores the necessity for increased supports for women and racialized people living with HIV. This paper details how HIV criminalization heightens, validates, and institutionalizes HIV stigma and argues that Canada should reform its legal approach to HIV.

### The context of HIV criminalization in Canada

Canada has been described as a world leader and hotspot of HIV criminalization [1,2]. Since 1998, there have been over 220 prosecutions for HIV non-disclosure in Canada [3], with the majority taking place in Ontario [4]. The most common charge is aggravated sexual assault, which carries a maximum sentence of life imprisonment and mandatory registration on the sex offender registry [5].

In Canada, the disclosure of HIV has been mandated by law since 1998. In R. v. Cuerrier, the Supreme Court of Canada ruled that persons with HIV have the legal responsibility to disclose their status if their sexual interaction could expose their partner(s) to “significant risk of bodily harm” [6]. In 2012, the Supreme Court of Canada revisited the non-disclosure of HIV in the companion cases R. v. Mabior and R. v. D.C. The court clarified the law, finding disclosure to be necessary when coupled with a “realistic possibility of transmitting HIV” [7].

There has been a general decline in prosecutions since their peak in 2010 [4], likely due in part to the advancement of prosecutorial policy over the past decade. At the provincial level, the last few years have seen the adoption of prosecutorial guidelines, directives, or instructions aimed at limiting HIV non-disclosure cases in Ontario, British Columbia, Quebec, and Alberta. In these provinces, there is a moratorium on prosecuting cases of HIV non-disclosure where the person living with HIV is on antiretroviral therapy and has maintained a suppressed viral load (of under 200 copies/ml) for four to six months (depending on the province), regardless of the type of sex involved [8].

At the federal level, in December 2018, the Attorney General of Canada issued a directive regarding the prosecution of HIV non-disclosure cases, which applies to the Northwest Territories, Yukon, and Nunavut. These prosecutorial guidelines state that PLWH will not be prosecuted when they are taking treatment as prescribed and have maintained a suppressed viral load (of under 200 copies/ml), or “generally” will not be prosecuted when condoms are used or when the partners only engaged in oral sex, unless other risk factors are present [9].

Following many years of community advocacy, in October 2022, the Federal Government of Canada undertook a public consultation on Criminal Code reform with regards to HIV non-disclosure [10]. In June 2023, the Department of Justice released a report summarizing the consultation feedback. According to the report, 85% of respondents agreed that the Criminal Code should be amended to separate HIV non-disclosure from sexual assault law [10]. Additionally, 50% of participants supported an amendment that would limit the law’s application to cases where the accused intended to transmit HIV, 50% agreed that the law should only apply to persons who actually transmit HIV, 61% thought the law should not apply if the accused took reasonable precautions to prevent transmission, and 59% opposed the creation of a new offence [10].

Although promising, these developments can be seen as uneven and insufficient. Advances in prosecutorial policy are vulnerable to the whims of future governments and, as they currently stand, do not apply to six provinces. Also, in the provinces, PLWH may still be charged when using condoms if they do not have a suppressed viral load. The public consultation, while welcome, has yet to translate into policy reform. Advocates and researchers caution that, as long as PLWH can be charged under criminal law, they remain at risk [11].

### The impacts of criminalization

The field of HIV criminalization has been built upon the work of PLWH and activists, public health and legal scholars, sociologists and criminologists, health care workers, and governmental and non-governmental organizations. Research on criminalization has examined the content and application of HIV laws across different jurisdictions, the impact of media representations of HIV criminal cases and its impact on prevention and the impact of HIV criminalization upon prevention initiatives and PLWH [12]. The literature is overwhelmingly critical of the use of criminal law to respond to the perceived risk of HIV transmission. Scholars question the effectiveness of criminalization as prevention and note the counterproductive and negative impacts upon both public health and prevention efforts [13–17]. Criminalization decreases testing among high-risk populations [18–20], within the population more generally [21,22], and reduces engagement with treatment and care [12,23–25].

Many issues stem from this legal approach. The law surrounding HIV non-disclosure is increasingly being used against those who are made marginal by structural conditions, such as Indigenous women, Black men, and gay men [26]. Despite representing 3.5% of the Canadian population, Black people represent at least 22% of those charged regarding alleged HIV nondisclosure [4]. Since the Mabior decision, 48% of people charged (in cases where race was known) were Black men [27], and the number of cases against gay men has increased [11]. While sexual assault laws were created to protect women from harm and exploitation, HIV criminalization leaves women vulnerable to (and disproportionately at risk of) prosecution [28–30].

Another issue with HIV criminalization is the law’s failure to keep in step with scientific and medical understandings of HIV and transmission risks [31,32]. PLWH are often indicted in situations that involve little to no risk of transmission. There is a firm and growing consensus in the scientific community regarding the inability of PLWH, who have attained an undetectable viral load, to transmit the virus to others [20,25,33]. Within the scientific and HIV communities, this is commonly referred to as ‘undetectable = untransmittable’ or ‘U = U’. The Supreme Court of Canada’s 2012 ruling that both condom use and a low viral load are required to avoid a “realistic possibility” of transmission is inconsistent with scientific evidence [34]. By failing to keep in step with medical developments, the law criminalizes interactions which involve negligible to zero risk of transmission. The disconnect between understandings of risk and transmission fuel HIV stigma [12], and complicate the lives of PLWH, particularly within their romantic and intimate relationships.

Among PLWH, there is considerable confusion, and anxiety regarding HIV criminalization [19], particularly with regard to what qualifies as ‘significant risk’ [16,23,30,35,36] or ‘realistic possibility of infection’. This is due in part to the evolving and uneven application of the law, and inconsistent rulings within different jurisdictions across the country [37]. Uncertainty regarding the law and one’s legal responsibility compromises the capacity of PLWH to make informed decisions and to navigate the law safely. This is especially pronounced for those who lack awareness of the law and have a higher viral load, due to systemic barriers that block access to HIV treatment and care [38].

Scholars have noted the inadequacies of legal attempts to contend with the intricacies of disclosure and sexual behaviour [22,39,40]. Although HIV criminalization impacts an individual’s perceived incentives and motivations for disclosure [41], the deployment of the law has not increased rates of disclosure [42,43] and may, in fact, discourage disclosure [44,45]. While some PLWH have altered their sexual practices in ways to decrease transmission risk and vulnerability to law [46,47], research has demonstrated little to no effect on safer sexual practices or condom use [48]. As Adam et al. note [48,49], HIV criminalization fails to acknowledge the complexity and fluidity of sexual decision making.

HIV criminalization is emblematic of a policy shift in the approach to addressing the HIV epidemic. Criminalization has been shown to have no effect on HIV incidence [50]. HIV criminalization exists within a grey area located between public health and the criminal justice system–a sphere which others have called the ‘medico-legal borderland’ [51]. Criminalization erodes public health norms generated through the ‘mutual (or shared) responsibility model’ [30,52,53], which assumes that sexual actors are self-empowered and self-motivated to engage in safer sex [54] and will ask their partner information about sexual and STI histories, and drug use, before sex [55]. Instead, criminalization draws from the ‘individual (or personal) responsibility model’, where PLWH bear responsibility for the behaviour of their partners [56]. The adoption of this model by policymakers decreases the mutual exchange of information and shared responsibility for safer sex between partners [57]. By creating the public expectation for PLWH to disclose, while simultaneously making disclosure riskier and more difficult [31,53], the law discourages HIV-negative individuals from taking responsibility for their own exposure to risk [58] and leaves them potentially vulnerable to situations where their sexual partners are unaware of their actual serostatus.

The law negatively impacts the lives of PLWH and their communities [31,42,51,59,60]. The law takes a toll on their mental health [61,62]. PLWH often feel vulnerable to the law, regardless of their sexual behaviour [43,63]. Criminalization contributes to fear of secondary disclosure [57] and makes it more difficult to live openly with one’s HIV status [63]. The law can dissuade PLWH, particularly women, from accessing police services [64].

The law complicates the relationships of PLWH through the enabling of concerns regarding potential prosecution, false claims of non-disclosure, the capacity to provide evidence in court, as well as violent reactions from partners after disclosure [57,65–67]. Criminalization redefines consensual sex [68] and contributes to a perceived one-sided burden of PLWH responsibility for safer sex [69]. The literature notes various strategies PLWH utilize to navigate criminalization within their relationships. Strategies observed to protect self from accusations under the law include anonymous sex without disclosure and abstinence [40,70].

HIV criminalization has led to some PLWH to engage in self-surveillance–through the documentation of their disclosure practices. Kilty & Orsini found that AIDS Service Organization (ASO) workers advise their clients to document their disclosure by disclosing in front of someone (friends or professionals), disclosing publicly (posting status online or over social media), by creating a paper tail (through text messages, email, or saving recordings), and through the use of informed consent documents [71]. French found some counsellors advise clients to also document their partner’s reaction, in order to be able to prove that their disclosure was understood [72], despite an awareness that the most meticulous documentation methods may not protect their clients from the law. While other scholars have noted tales of disclosure documentation in the context of examining other topics–for instance, by saving online chats [65,69], disclosing in front of someone [73], or profiles in dating apps [74].

### HIV disclosure

The decision to disclose one’s HIV status is multifaceted and can be complicated by contrasting desires, concerns, and priorities. Many PLWH engage in a cost-benefit analysis where they weigh the pros and cons of disclosure [75–77]. Disclosure decisions have to be made repeatedly, can change over time [41], can vary by relationship type [78], and can have an impact on one’s identity [79].

Disclosure is motivated by a desire to protect one’s partner [80,81], a sense of duty and responsibility [71,75], a desire to facilitate connection [77], and to acquire emotional, physical and social support [82]. Disclosure decisions can vary based on previous reactions to disclosure [83], relationship status [84], context [85], residence and demographic factors (such as age, sex, race, ethnicity, and SES) [41]. In addition to criminalization [49], HIV disclosure is complicated by fears and concerns regarding rejection [86,87], HIV stigma and discrimination [88–90], intimate partner violence [91], abuse [78], abandonment [92], privacy and secondary disclosure [93], a loss of economic support [94], and feelings of shame [95] and self-blame [77].

Disclosure can lead to many benefits for PLWH. Disclosure can facilitate greater access to treatment and adherence to medication [76] and better health and treatment outcomes [96]. Discussing one’s HIV status can strengthen relationships [75], prompt partners to get tested [76], lead to increased discussion of safer sex options [97], and can motivate condom use and safer sex [98,99]. Disclosure is also associated with various affective and material benefits such as enhanced wellbeing and mental health [100], greater self-efficacy [101], feelings of relief [102], and increased access to social, financial, and psychological support [76,86,90,103–105]. In contrast, through experiences of HIV stigma, disclosure can lead to negative impacts, including regret [73], social isolation [87], concern regarding loss of employment, discrimination, and rejection [71], as well as poor mental health outcomes (increased anxiety and stress) [106,107].

Research indicates that non-disclosure can lead to psychological distress [106–108], maladaptive coping strategies [100], and poor adherence to medication (which is associated with higher viral loads, and poor health and treatment outcomes) [109]. Non-disclosure is impacted by one’s occupation, relationships status and length [108] as well as support network [84].

Disclosure concerns and practices are differently nuanced for women living with HIV (WLWH). Women are less likely to disclose their HIV status than men [110]. Before sharing their status, WLWH often evaluate the potential consequences and benefits of disclosure [78]. In a recent study, Patterson et al. found that the majority of WLWH in their sample (over 75%) feared HIV disclosure [111]. Many have concerns about secondary disclosure (non-consensual disclosure of their status to someone) [112].

Disclosure is different for racialized PLWH than it is for white PLWH. Black MSM in the US, for instance, are less likely to disclose than white MSM [113] and fear that their status could be used against them in their communities [114,115]. Among Black MSM living with HIV, disclosure can result in violence and rejection from friends, family, and partners [116], a loss of housing and employment, temporary or permanent alterations to social and familial relationships, as well as suicidal ideation [117].

### Relationship to literature

This paper complements the literature by providing a robust account of the impact of HIV criminalization upon the everyday lives of a diverse group of PLWH, and by providing more evidence supporting need for legal reform. This research adds to the HIV criminalization literature by detailing how the impacts and unintended harms of this legal approach are patterned along lines of gender, race, and sexual orientation. This paper extends the disclosure literature to consider, in detail, the navigation and documentation strategies of PLWH, and how access to these strategies varies in terms of social location. This research also takes a strengths-based approach, through highlighting the resilience of PLWH through their innovative responses to legislation and the steps taken to mitigate its impact within their lives.

This study helps to address noted gaps surrounding the perception and experience of the law by PLWH [118], the impact of the law upon PLWH and inequality [65], the awareness and understanding of the law among PLWH [35,43,53], the impact of the law upon testing and treatment [119], and means to improve disclosure techniques [106]. To this researcher’s knowledge, this study is the first to examine this criminalization and the impact on relationships, using qualitative methods, since the law surrounding HIV non-disclosure changed in 2012, or to examine the disclosure documentation strategies PLWH create and employ to navigate vulnerability to the law.

## Methods

### Ethics statement

This study’s research protocol received approval from the HIV Research Ethics Board at the University of Toronto (Protocol Reference # 29397). After reading the study FAQ sheet (which served as a consent form), participants provided verbal informed consent, which was recorded and noted in the transcript. This process was one of many steps taken to limit both the use of personal identifiers in the study and the evidentiary worth of the data, should the records be subpoenaed during a potential HIV non-disclosure investigation.

### Intersectionality

This paper employs an intersectional lens to gauge the impact of HIV criminalization upon PLWH and to examine how these impacts (and access to responses) vary in terms of gender, race, and sexual orientation. Coined by Crenshaw, intersectionality is a sociological framework that examines how aspects of one’s identity intersect, and how these interact with social structure to shape experiences of privilege and oppression [120]. Within intersectionality, race, class, and gender are considered to be mutually constitutive and inextricably linked [121]. This framework can also be used to interrogate other layers of difference, including sexuality, nationality, religion, and ethnicity [122].

Intersectionality arose to address the lack of representation of Black women in academic, intellectual, and political thought, and sought to examine how gender inequality was constituted in relation with class and racial inequalities [123]. A key aspect of intersectionality is that difference is not additive–meaning, for instance, that one cannot add the experiences of a white woman to those of a Black man to adequately estimate or reflect the experiences of a Black woman [124]. Intersectionality facilitates the awareness of how social location and identity shape lived experience and how, through interaction with the hierarchies within social institutions, can restrict or facilitate access to power, representation, and resources.

Intersectionality is a versatile methodology that enables researchers to access representative accounts of lived experience, and to examine how individuals interact with (and are impacted by) social institutions, along various lines of social difference. Intersectionality allows researchers to look at social structure from the perspective of the subordinated and is well suited to demonstrate how systems (such HIV non-disclosure legislation) impact both the experiences of individuals and groups, while also providing an analytical means to access these impacts (and responses) across various levels of difference (such as race, gender, and sexual orientation).

### Methodology

This qualitative study is based upon one-on-one, in-depth, open-ended, semi-structured interviews with 54 people living with HIV. This research draws upon qualitative methods to facilitate a deeper understanding of the context in which people living with HIV make their decisions regarding disclosure, how they experience criminalization, and how the law impacts their relationships, daily lives, and communities.

Study participants were recruited through outreach to ASOs and HIV Health Clinics across Ontario, and through snowball sampling. Recruitment materials took the form of an information poster and FAQ sheet. Participants were offered $35 in remuneration for their participation in the study. Recruitment ran between February 15^th^, 2014 and February 13^th^, 2016. No minors participated in this study. The author received no specific funding for this study.

Interviews ranged from 40 to 120 minutes in length, with most being in the 60-to-70-minute range. The interviews, conducted by the researcher, were digitally recorded, and took place in person either in a private location or over the phone. Each participant chose a pseudonym during the interview process to increase their own confidentiality. The interview guide, which consisted of questions phrased in general grade 8 level English, evolved throughout the data collection processes, with participants’ thoughts and feedback informing the shape of questions for future interviews. No participants withdrew from the study or opted to not participate after reading the consent form. Part way through the data collection process, a pre-screening questionnaire was adopted to facilitate increased representation from under-represented age groups.

The methodology of this study was informed by a pilot study in which 21 HIV positive gay, bisexual, and other men who have sex with men (GBMSM) were interviewed about their experiences with disclosure, sexuality, condom use, relationships, and criminalization. Although the data from the pilot study was rich, preliminary data analysis motivated methodological changes involving the broadening of recruitment and reformulation of the interview guide (to include topics and themes of community, human rights, impacts of the law, ASOs, and prevention). The 21 pilot study participants made important contributions which enabled the main study’s diverse group of 54 positive straight and queer women and men to share their stories and experiences about their lives under criminalization.

Women comprise 24% of the sample (13/54), and men 76% (41/54). 41% of participants self-identified as being members of a racialized community. With regard to sexuality, 41% self-identified as being straight and 59% as gay, bisexual, queer, or two-spirit. There was relatively balanced participation in terms of participant age, with respondent ages ranging from the late teens to late sixties. A spectrum of education levels was represented, with formal schooling ranging from a grade 2 education to post-doctorate degrees. Many participants were single, while others had been with partners for up to 30 years. People from 20 towns and cities took part in the study, drawing upon a range of rural and urban experiences. There was little variation in terms of income with 79% of the sample living below the poverty line of $25,000. There was a great variety with regard to time living with HIV. Some respondents had been diagnosed with HIV for weeks or months before their interview, whereas others had known about their HIV status for over 30 years. When combined, the participants in this study have 661 years of experience living with HIV.

The interviews were transcribed by hand and organized using the qualitative research software NVivo. All unintended disclosed identifiers were altered throughout the transcription process. Codes and coding approaches evolved throughout the data analysis process. Each interview was coded via thematic analysis, drawing from the guiding principles of grounded theory (Strauss & Corbin 1998) which allow for conceptual understandings and relationships to emerge from participants’ accounts and their own interpretations of their experiences. Interviews were initially coded with attention to preliminary and a priori codes. Grounded codes emerged from the data via open coding, axial coding, selective coding, and line-by-line coding practices. Throughout the coding process, attention was paid to patterns and intersections within the data.

The everyday realities of living with HIV vary considerably in terms of sexuality, gender, race, class, age, education, and geographic area. Living with HIV is different for a young, white, middle class, gay man living in a metropolitan city than it is for an older, working class, straight, Indigenous women, living in a small town with three kids. By focusing on a diverse group of PLWH, this study provides a robust picture of the impact of the criminalization of non-disclosure upon the relationships and navigation strategies of PLWH across Ontario. Through recruiting a diverse sample, the study is able to highlight differences and commonalities among the lines of race, gender, and sexuality, providing an intersectional view and representative voice of what it is like to live and love with HIV under criminalization in Canada.

## Results

### Impact on relationships

HIV criminalization impacts the relationships of PLWH in a variety of ways. PLWH in the study conveyed concerns with the law’s impact on their dating desires and practices, the power imbalance the law enables within relationships with serodiscordant partners, the privacy implications of being investigated or charged, the law’s potential for exploitation, as well as their potential treatment by both police and media.

PLWH have the same sexual and romantic needs as everyone else. Within their interviews, participants expressed interest in sex, companionship, emotional connection, partnership, love, and family. They want to share their lives with someone and want to be wanted. These desires, however, are complicated by criminalization.

*But you know, I’d like to marry a woman, and I’d like to have kids and an independent life, so that I don’t want to do all this shit alone. Now I don’t know if I’m able to have, uh, marry or something to find the same category human. And how do I find someone? I talk to many people on medication, too. But nobody’s healthy. <Pause> And at the end of the day, I feel lonely…*

–Mark, early 20s, bisexual Asian man, single, living with HIV for 7 months

Among participant accounts, there is a prevalent concern that one’s HIV status could be used against them by a spiteful former or potential partner. Many worry that a partner could threaten to tell the police that they had not disclosed, regardless of their actual disclosure, or sexual behaviour. By placing the responsibility for disclosure (and sexual health) solely upon PLWH, the law creates a power imbalance within relationships where negative partners, by default, have more power within the interaction and relationship. The decrease in bargaining power, enabled by the law, can leave PLWH open to potential abuse by potential, current, and former partners (through blackmail, extortion, and/or control), as well as the criminal justice system (through false claims of non-disclosure).

*I think the law opens a lot of vulnerable situations for someone that is living with HIV. You never know, for example, nothing guarantees that if I decide to break up with my boyfriend who knew for 3 and a half years that I was positive that he can’t go press charges against me. All the sudden I am the guilty one and I have to prove my innocence. No matter what the situation is, it would always put the HIV positive person in a vulnerable situation. I know in fact of someone in Quebec who was in an abusive relationship, and the person knew about her status, not from the first day of the relationship, but the person knew—and because the HIV positive person pressed charges against her partner because he was physically abusing her, she ended up being the one charged because the guy went to the police and said, “Oh, she is HIV positive but she just told me a week after we had sex.”*

–Andy, late 30s, gay Latino man, partnered, living with HIV for 9 years

Criminalization exacerbates the privacy concerns of PLWH. The idea of being charged with or investigated for a potential case of non-disclosure prompts great stress and anxiety. Beyond the possibility of jail time, the fear of being subject to the attention of law enforcement stems from the resulting violation of privacy. This results in a person living with HIV being tacitly ‘outed’ as having HIV within their community, which can expose themselves, and those they care about, to stigma and discrimination.

*Disclosure is a big issue for women who are seriously looking for someone to have a relationship with, because they think they’re just going to turn them off. There’s a lot of ignorance about HIV out there. Being very careful about who you tell, too, because then they might tell other people. Women are really much more prone to think about disclosure in those terms. They want to have control in the situation. Especially African women who fear stigma within their communities and fear rejection in their community because they feel like they really need their community here. And word getting back home, too. That’s a big issue for African people. Not just for women, but for men, that it’s going to affect their status within the community. A lot of that is really because people are kind of ignorant about what it means to be HIV positive.*

–Judith, late 30s, straight Indigenous woman, living with HIV for 5 years

Many PLWH in the study were concerned about how the law can make them vulnerable to exploitation through false claims of non-disclosure. Some had concerns about their disclosure being used against them by a casual or romantic partner, or by someone in the community. HIV criminalization can expose PLWH to threats of blackmail and violent reactions from partners.

*The building I live in is the only building in Toronto that is fully HIV positive. You have to be HIV positive to live in the building. Most of the community downtown knows this. I remember once I picked up a boy and took him back. He started saying he was going to call the police, unless I gave him money. He was going to call the police and say I hadn’t disclosed, when I had. He hit himself in the head and started yelling, “Rape!” at four or five in the morning, just to get money.*

–Lore, late 40s, gay Asian man, living with HIV for 27 years

Criminalization also exacerbates concerns regarding potential treatment by police and media. Many in the sample believe that, should they be charged, they would not receive equal treatment by the criminal justice system or the media. PLWH feel that they are seen as being guilty by default. This can lead some to not access police or social services. The criminalization of HIV nondisclosure frames HIV as a weapon and PLWH as sexual predators. These themes are perpetuated and disseminated by the media.

*I know someone who hasn’t had sex for ten years because she kissed a guy—she made out with a guy—and after they made out, she told him that she has HIV. He made her leave and burned the sheets. So, because of that… <sigh> He called the police and they interrogated her for ten hours. Ever since then, she hasn’t wanted to have sex at all.*

–Brooklyn, early 30s, two-spirit Indigenous woman, partnered, living with HIV for 5 years

### Navigation strategies

Among PLWH in the study there is an awareness that following the law (and disclosing when not using a condom and having a low viral load) may not be enough to protect themselves from the criminal justice system. PLWH invest much thought and energy in contemplating ways to contend with (and protect themselves from) the law and to minimize the impact it has upon their sexual and romantic relationships. These navigation strategies include staying in relationships, celibacy, serosorting, anonymous and casual sex, leaving the jurisdiction, universal disclosure and, somewhat counterintuitively, non-disclosure. Additionally, PLWH employ an array of different methods to document their disclosure, in hopes of providing evidence if called to court.

Many PLWH modify their approach to relationships in direct response to the law. For some, HIV criminalization functions as an external pressure to stay with their partner. Many partnered respondents mentioned that, should their current relationship end, they would not date again out of concern for the law. Others mentioned staying in unhealthy and abusive relationships as that was better than having to contend with the threat of a non-disclosure charge, or disclosure and dating.

*Does the law affect my own relationships? Yeah, it does. I’m a little afraid every time I have an argument, I’m always a little afraid that if something were to happen where police were involved, if that came up, even if I was the person who was being compromised, I’m instantly wrong so therefore I should probably keep quiet and put up with more abuse.*

–Judith, late 30s, straight Indigenous woman, living with HIV for 5 years

Other PLWH avoid and protect themselves from the law, and the implications of being charged, by foregoing romantic and sexual relationships altogether and living single and celibate lives. The majority of the sample reported having less sex than they would if HIV were not criminalized.

*A lot of the women I know have been celibate for twenty years. They’ve been celibate since they’ve been diagnosed. They’ve decided they just can’t have sex, since they’re positive. I would hope that things have changed. I think that some are still in the old mentality that they could infect, so they don’t want to, not realizing that it’s not like it used to be. I do think the law has a huge impact on PLWH. It makes them think it’s still as bad as it used to be when it’s not.*

–Jade, late 20s, straight white woman, married, living with HIV for 6 years

Some PLWH contend with the difficulties of dating under criminalization by only pursuing sexual or romantic relationships with other people living with HIV. By only dating other PLWH (a phenomenon also referred to as serosorting), participants feel that they can circumvent the potential issues of rejection, stigma, and vulnerability under the law (false claims of non-disclosure). Many participants indicated that they would not consider relationships with people who were negative and prefer to only pursue relationships with other positive people. For PLWH in the sample, a core benefit of the serosorting navigation strategy is the protection it affords from the law (as concerns regarding potential blackmail or victimization are rendered moot by removing the power imbalance between partners).

*I’m in a relationship right now. We’re both positive and we started out as friends as kind of like support for each other. That was a non-issue. I would disclose definitely if it was like, “let’s do drinks,” because there’s no point in going on a date with someone unless they know right from the get-go. I wouldn’t want to waste my time if he couldn’t see themselves in a relationship with someone who’s positive. The majority of the people I’ve been sleeping with the past six months have been positive, so it’s kind of always been like, I’ve sought them out on apps. It’s safer.*

–Kyle, late 20s, gay white man, partnered, living with HIV for 6 months

Some PLWH, particularly GBMSM have adapted to sex and dating under criminalization by engaging in anonymous sex. Within certain public sex environments (like bathhouses, parks, or darkrooms in bars), non-disclosure is common. Based upon participant accounts, in these contexts, it is considered to be the responsibility of each individual to use condoms, or to inquire about HIV or STIs, should they be concerned. Not following this practice can be interpreted by participating PLWH, as an assent to any risk that may be present, or an indication that the individual is also positive.

*In a bathhouse, I think that it’s seldom discussed. Especially in open public rooms. When you get into other scenes, I think it’s a little better. You know, like bar scenes and stuff like that. You know, you’re going to see those people again on a regular basis. So that I find that it’s not so hush-hush. But, in the bathhouses or sex parties, for sure. It is a totally different story. Like, if somebody came over and, you know, it was a blow job and we fooled around, and that was that. And I wasn’t going to see them again. Yeah, then I wouldn’t disclose. When it comes to the point that they start to see them more and more, then I disclose.*

–Ben, early 30s, gay white man, single, living with HIV for 1 year

Engaging in anonymous sex can be an effective means to navigate HIV criminalization, in so far as, if partners cannot identify or contact one another, then it will be difficult to make a claim of non-disclosure. As Ben describes, disclosure can be linked to familiarity. When sex moves from anonymous to a more casual relationship, where partners can identify and contact each other, then disclosure is more likely to occur. As Kilty and Orsini note (2019b), some ASO workers recommended that their clients pursue anonymous or casual sex if they do not want to disclose to others. These interactions may be further impacted by the recent increased access and uptake of pre-exposure prophylaxis (PrEP)–a highly effective medication regime that can be taken preventatively by HIV-negative people to reduce their chances of acquiring HIV from sex or injected drug use.

A few participants mentioned the navigation strategy of leaving the country to have sex. By only having sex in jurisdictions where Canadian law does not apply, PLWH can disclose or not disclose free from the threat of legal recourse or exploitation under the law (via false claims of non-disclosure).

*I don’t have sex because of these laws. For me, I find for me to have sex, I have to do it outside of Canada when I go to the Caribbean. With the laws here I don’t want to get myself into trouble. I was homeless for eight years before I moved to Canada in 2008. When I came here, I found out during the refugee process that I was HIV positive. I don’t want to get deported to my home country. For me, when I have sex, it is in the Caribbean.*

–Vince, late 20s, gay Black man, single, living with HIV for 8 years

Just thinking about all that legal stuff, and the stigma… So, I understand there’s a lot of people who want to have a baby. And they’re undetectable. So they go to whatever country, whether in Europe or somewhere, where they can possibly have sex because their levels are low and they’re not disclosing without being threatened or charged—that kind of risk. Get pregnant, come back here to have their baby and there you go. Everything is gravy. I know people who have done that. … People are terrified. There’s a lot of women who are very maternal and want to have babies, or have babies already and think that they can… But I had to go to Georgia because it got so complicated. It’s painful.

–Tara, mid 50s, straight white woman, single, living with HIV for 30 years

Some PLWH protect themselves from HIV criminalization by being open with their HIV status. Universal disclosure occurs when a person living with HIV is largely open with their status with the majority of people in their lives. By living publicly with their HIV, some PLWH believe that, should they be accused of non-disclosure, they would have a long list of witnesses (ranging from friends, family, coworkers, and former partners) who could testify as to their propensity to disclose. Although some feel that this navigation strategy affords great protection from the law, it can be a difficult path to take as it can open one up to increased HIV stigma and discrimination in their daily lives.

*I’ve gone back and forth on this a million times. To be honest, like last month I made… no one in my life really knew, only close people. Probably ten people in my life knew, and that’s nobody that I personally disclosed to, it was including like my doctors who said ‘You better keep this secret.’ It wasn’t until last month and I created a Youtube video and kind of told everybody, so now all my friends and family know, but I think, for me, if I were to disclose… I do have really good doctors and social workers and I have talked to them about if I got into a relationship and don’t feel like I could find the correct words. I asked if I could bring somebody in and they said it’s happened plenty of times. But, if there was a problem, since I have the video online, I think I should be okay. Now I’ve disclosed to everyone.*

–Teresa, early 20s, straight Black woman, single, living with HIV for 21 years

Some PLWH protect themselves from the law by following its precepts and disclosing their HIV to their potential sexual and romantic partners when necessary. Others protect themselves from the law, somewhat counterintuitively, by not disclosing their status at all. For these individuals, non-disclosure is seen as a means to protect oneself from the risk of complying with the law (potential charges and/or exploitation via the legal system due to someone knowing their HIV status). This approach was particularly common when individuals had an undetectable viral load or were practicing safer sex.

*If you disclose to an individual and they say, ‘Oh, it’s okay. We’ll use a condom and that’s fine,’ because they had been educated by ASO and posters that, you know, I’m undetectable, they are not going to get it. But then they go off and somebody else doesn’t disclose. So, you’re the good person. You’ve disclosed to them. They’ve had sex with you. Now they’ve gone off and the bad person does not disclose to them. Four, five, six months down the line, they’ve had sex with 20 odd more people, unprotected because, ‘Oh no. He didn’t have HIV.’ You go and get tested. You find out that you have HIV. Who do you go back and attack via the legal system? It’s the positive person you know. It’s like, ‘Oh, I had sex with that positive guy. Who was he? Oh, here’s his emails.’ BAMB! None of those people that did not disclose are as easily found and are going to court.*

–Jasper, late 20s, gay white man, single, living with HIV for 1.5 years

### Disclosure documentation

A common strategy to contend with the realization that disclosure itself is not enough to offer protection from the law is to document their disclosure, in some form. By documenting their disclosure, PLWH feel that they will be able to prove that disclosure happened and defend themselves against allegations, in court or with the police, should they be charged or investigated. The strategies by which PLWH document their disclosure are quite varied and are a form of innovation and resilience. Some will disclose in front of others. Some will keep records of conversations. Some will ask partners to sign waivers. Some will keep detailed histories of their sexual interactions. Some will keep and label used condoms.

A growing disclosure documentation technique is the utilization of waivers. Some PLWH will ask potential partners to sign a waiver confirming that they have disclosed their HIV status before a sexual encounter occurs. Others will take the waiver a step further and have witnesses watch the signing of the document.

*Yeah, like some people are a bit scared of the law. So, they do things to protect themselves from it. So, for instance, the law now says you have to use a condom, like if you are not going to disclose you have to use a condom and have a low viral load. So, they’ll protect themselves from the law by making sure they have proof they used a condom or proof that their viral load is low, that kind of thing. I’m not saying you have to do that. But some people do things to, some people will disclose but then they don’t want it to turn into like he-said, she-said thing and so they’ll get them to sign something saying they disclosed. Right, so there’s proof. So, some people do stuff like that. Yeah, I think, yeah. I think for me, that would be it.*

–Lewis, mid 40s, straight Black man, single, living with HIV for 4 years

Some PLWH will disclose or re-disclose their HIV status in front of witnesses like friends or family. This way, these people could be called to testify, should there be an allegation of non-disclosure. Others will bring partners to HIV-centric events such as workshops or social gatherings run by AIDS Service Organizations. While ensuring there are multiple witnesses who could testify if required, this strategy has the added benefit of educating the partner about HIV and getting them connected with the HIV community. As Jack explains, while discussing the impact of the law on disclosure practices,

*For me personally, no. But for my friends, yes. Actually, now, the three friends who have actually gone to prison now only sleep with positive people. So, yes and no. Yes, it’s making more people disclose. And no, because a lot of times when they disclose, it still comes back and bites them. One of my friends who went to prison, his girlfriend—he told her he was positive, and she said he didn’t. So that’s the one who did 18 months for nondisclosure. So, like I said, yes and no. So, I tell all my friends, “Make sure you tell them in front of somebody who can back you up, or get them to sign a document.”*

–Jack, mid 50s, gay white man, single, living with HIV for 21 years

After disclosing privately, some participants will take their partners to see their doctor or public health nurse to further discuss their status, often before high-risk sexual activity occurs. This approach ensures that there is impartial and official documentation of disclosure, that can be subpoenaed and used as evidence. Others will use regular visits to the doctor to ensure that there is an up-to-date record of their viral load.

*Sometimes I’ll disclose in front of my doctor. I go in with my partner—sort of my regular medical thing where I go to my treatments—and I bring her with me, and I explain to my doctor, “This is my partner, and she needs to be told that I am HIV positive, and it has to be written down.” That way there is a witness that she knows, so she can’t go back later and be like, “You didn’t tell me.” It’s just a way to protect myself, basically. …*

–Smith, early 50s, bi Indigenous man, dating 1.5 years, living with HIV for 17 years

Other PLWH document their disclosure by recording conversations in which they are discussing their HIV status with their partners. These conversations can be in-person, over the phone, or online, often without their knowledge. When using a digital recorder or smartphone, many will email backups of the interchange to themselves in hopes of ensuring the file’s safety.

*My ex, he threatened me. We were in a fight and he was like ‘I’ll call the cops and say you didn’t disclose’. I was like ‘Fuck you!’ and showed him texts I sent about picking up my meds. Said he’d get in shit for making a fake charge. I don’t know what woulda happened if I didn’t have those texts. <pause> I’m with a good guy now but, yea… I’ve saved our texts too.*

–Jayli, late 20s, bi Indigenous woman, partnered, living with HIV for 10 years

Some PLWH keep a disclosure history. This refers to a type of journaling device in which PLWH record a detailed account of their disclosure practices, replete with specifics such as how they disclosed, how the partner reacted, any witnesses, subsequent disclosures, pertinent dates and so forth. The logic behind a disclosure history is that, should one’s disclosure be called into question, the individual would be able to provide the court with a clear and robust account of other verifiable disclosures.

Some PLWH take a more digital approach to disclosure and documentation. Many will disclose their status within their dating profiles. They believe that by having their HIV status public within the app, chances of a non-disclosure charge will decrease. Online disclosure has the added benefit of being easy to record and document.

*It happens, and usually electronically. It’s on my profile, I’ll ask people if they’ve read my profile—it’s obviously not a problem for you if you want to connect. I’d rather save people the time and the effort. There are sometimes when it’s like, “I really kind of like you, I’d rather meet you—and before we fuck, I’ve got to tell you I’m positive. Oh you are too! Oh, okay, great.” Or whatever case. Most of the time the conversation happens before we even meet. And that just comes from years of wanting to make it easy for everybody- and a fear of rejection…. For a couple of years, it became kind of an art*.

–Stephen, mid 30s, gay white man, single, living with HIV for 16 years

Others will disclose, not in their main public profile, but through in-app conversations or via text message. Some will take screenshots or videos of these conversations. Others will, after disclosure in person, mention their HIV status or re-disclose over a text message, email or over a messaging service. Digital logs of disclosure are felt to be of increased evidentiary worth due to their built-in date-stamp.

*I think that in one way, the law is the law, but in another way, people have found a way to kind of push back. But it still comes back to bite you in the ass if someone doesn’t like you. [laughs] When I have conversations with people, it’s hard when you do it in person, like at a bathhouse or whatever, but online, one of the things we have talked about is how it keeps a record of the conversation with the person that indicated that you disclosed. And also keep a copy of your profile indicating that you do have your status listed. So that way if the person does say, “Hey, you never told me,” it’s like, “Well, obviously it’s on my profile, yes I did, here’s the conversation.”*

–Trixie, late 20s, queer white man, dating 1 year, living with HIV for 12 years

Some PLWH will go to great lengths to document their disclosure, such as saving condoms.

*People can be kind of innovative about how to deal with this. People don’t want more trouble in their lives, and the idea of going to jail for sex… It turns people off so much that they don’t want to have to even worry about it. The other thing is that people then worry about how they can prove disclosure, because people have found that if they have a breakup that wasn’t pleasant, they’re being accused of not disclosing until after they’ve had sex. Women will talk about taking that person to their doctor with them so that they have a record, so the doctor can say that this person was in the room when they were talking about their HIV health. One person said he kept the condoms in his freezer. People go to incredible extremes to prove disclosure because they feel like they might need to do that eventually. I can’t imagine my freezer full of used condoms.*

–Diane, early 60s, straight white woman, married, living with HIV for 7 years

As Diane mentions, a peer saves condoms (named and dated) in the freezer in order to prove that they were used with their partners, just in case one accuses them of non-disclosure.

## Discussion

### Navigation strategy effectiveness

Although innovative, there are many limitations to the strategies PLWH employ to navigate the law, protect themselves and document disclosure. From a legal perspective, many of these approaches can be problematic and of dubious evidentiary worth. For instance, for waivers to be effective, they should be witnessed and signed by others and the signatories should be sober. Audio recordings without consent, although legal in Canada (under Section 184 of the Criminal Code), may be illegal in other jurisdictions. Although taking a partner to see a doctor or public health nurse may be an effective means of documenting disclosure, this approach does not fit for casual dating or anonymous type sexual interactions. This approach is also not necessarily accessible for PLWH who have trouble accessing health care professionals.

Digital disclosure has drawbacks as well. Digital records are not as secure as many believe. Computers can break, phones can be lost, and backups can fail. Accounts and profiles can be hacked or deleted. Digital images, emails, and tests can be deleted, corrupted or argued to be altered. Even the best disclosure documentation may be vulnerable to a technologically adept disgruntled ex-partner.

With disclosure, online or otherwise, the documentation of disclosure itself is not necessarily enough. One needs to demonstrate that their partner both received and understood the message. The problems with disclosure and the documentation of such can be frustrating for PLWH. They feel as if the system is designed for them to fail and that, whatever they do, it may never be enough to protect themselves from the law.

### Intersectionality

While PLWH have created navigation strategies to mitigate the impact of the law upon their relationships and lives, adoption of these strategies is uneven and is impacted by one’s social position. Based upon participant accounts, access to these strategies varies along lines of race, gender, and sexual orientation.

Women in the study, for instance, were particularly concerned with privacy, the violent reactions to disclosure and the potential exploitation enabled by the law. Women commonly reported navigating the law and its impacts through staying in relationships, and celibacy. While some women engaged in universal disclosure, HIV stigma was a primary concern. Women were particularly anxious about the impact of stigma on their family members and children. Serosorting and anonymous sex were not mentioned by women in the study, while non-disclosure was only reported by those who had a histories of sex work. Racialized women reported leaving the country to get pregnant. With regard to disclosure documentation, women were more likely to disclose in front of witnesses, take their partners to see a medical practitioner. Some women would save digital records of disclosure, like text messages.

Women do not have access to the same subcultural norms (which sanction anonymous sex), institutions (like public sex environments), technologies (like gay hookup apps that facilitate anonymous sex and online disclosure), population HIV epidemiology (that facilitates serosorting), or community-level awareness and history of activism (that supports universal disclosure). As such, women do not have meaningful access to the same spectrum of navigation strategies as GBMSM living with HIV.

Black, Indigenous, and other racialized PLWH in the study were more likely to express concern over privacy and treatment by police and media. With regard to navigation strategies, racialized PLWH mentioned staying in relationships, celibacy, and leaving Canada to have sex. Racialized men mentioned non-disclosure, whereas racialized women mentioned leaving the country to get pregnant. Universal disclosure was rare for racialized PLWH, due to perceived high levels of HIV stigma in their communities and a lack of community support. Serosorting was only mentioned by queer racialized men. In terms of disclosure documentation, racialized PLWH mentioned disclosing in front of witnesses, and taking partners to a doctor’s visit. Queer racialized PLWH also mentioned digital disclosure and having partners sign waivers.

Racialized PLWH often have to contend with high levels of HIV stigma in their communities, as is common in communities with traditional, conservative, or religious values. Due to experiences with HIV stigma, racialized PLWH feel an increased pressure for privacy. This helps to contextualize the preference for non-disclosure, leaving the jurisdiction, and celibacy. While queer racialized PLWH expressed having more options, straight racialized PLWH were left with fewer supports and ways to navigate the impact of the law upon their relationships. Beyond having access to fewer navigation strategies, concerns with privacy and stigma limit their capacity to disclose and curtail their access to HIV community support and medical treatment (both of which can contribute to decreased health outcomes).

GBMSM expressed concern with the power imbalance the law creates between partners. GBMSM were more likely to navigate the law through engaging in serosorting, anonymous sex, and non-disclosure. Across the study, GBMSM were most likely to engage in universal disclosure. Those GBMSM who mentioned leaving the jurisdiction were from racialized communities or had past experiences with criminalization. Other GBMSM mentioned periods of celibacy and staying in relationships. With regard to disclosure documentation, GBMSM were more likely to have partners sign waivers, record conversations, engage in digital disclosure, disclose in front of witnesses, create disclosure histories, engage in universal disclosure, or save condoms. Straight men, in contrast, mentioned staying in relationships, celibacy, and non-disclosure, and documenting disclosure through disclosing in front of witnesses.

GBMSM have access to the 2SLGBTQIA+ community. While people who stray from sexual and gender norms have likely always existed, the 2SLGBTQIA+ community as we know it in the West today was shaped, in part, by the AIDS crisis of the 1980s and 1990s and, as such, queer culture often involves a familiarity with HIV and sexual health. This subculture has incorporated understandings of sexual health and risk that center around a personal responsibility for health. Due to advances in treatment and advocacy, HIV is now considered by many to be a chronic manageable disease. As such queer subculture often sanctions non-disclosure in contexts where the potential for risk is high (as it is seen as a personal responsibility to protect oneself from risk via the use of condoms or preventative medications (like PrEP and PEP)). Similarly, queer culture celebrates sexual autonomy and has access to institutions (like bars and bathhouses) and technologies (like queer hookup apps) that facilitate anonymous and casual sex and include HIV status in their profiles. GBMSM who adopt the universal disclosure strategy have more community support due to the history and familiarity with HIV in the queer community. This strategy can be facilitated by the anonymity afforded to those living in larger metropolitan areas and is considerably more difficult for those living in smaller towns and rural areas.

The organization of sexual practices and sexual relationships within urban gay communities creates meaningful differences for how gay men orient their sexual practices, romance, and relationships. This facilitates their navigation of the challenges posed by criminal law. Straight women and racialized people living with HIV do not have access to the same navigation strategies and, consequently, experience more difficulties with life and love under criminalization.

## Application

While awareness of PLWH’s concerns regarding criminalization is growing in the literature, few studies have explored its impacts upon relationships, while fewer still have explored the strategies PLWH have created to navigate the law and document their disclosure. This study adds to the literature by displaying a thorough representation of HIV criminalization’s impact upon the relationships of PLWH, while highlighting the breadth of approaches and strategies PLWH have created in response.

With regard to HIV criminalization, despite displaying awareness of the law, some PLWH in the study expressed confusion as to its requirements (which is in line with the literature) [20,26]. Others, however, had a clear sense of the criminal legal requirements around disclosure and developed specific strategies to navigate the law based on this understanding. The study echoed the finding of PLWH feeling vulnerable to the law, regardless of their sexual behaviour [23,63], and corroborates research that notes how criminalization hampers PLWH’s ability to live openly with their HIV status, and can diminish their quality of life [67,69]. Participant accounts resonate with the observation that the law can discourage disclosure (in efforts to protect oneself from a potentially vindictive partner) [23,42]. This work confirms the observation that the law has shifted the burden of proof for disclosure onto PLWH [57], as well as concerns regarding potential legal exploitation by partners, and treatment by authorities. This study expands upon these findings by considering the strategies PLWH use to navigate these concerns, the role that gender, race, and sexual orientation in navigating the law, and examining PLHW’s views and reactions to HIV criminalization after the law was clarified by the Supreme Court in 2012.

This study details the broad harmful effect of HIV criminalization from the perspective of those most directly impacted by this policy, adds to the chorus of research which calls for the revision and repeal of the law surrounding HIV non-disclosure in Canada [3,19,47,57,125], and supports the community-based perspective that HIV-related prosecutions be reserved for cases in which there is actual, intentional transmission of HIV [11].

In addition to contributing to legal reform, these findings could be mobilized by healthcare and advocacy providers to create population-specific initiatives, intended to support PLWH as they learn how to protect themselves from the law and navigate dating under criminalization. These initiatives should take a strengths-based, trauma informed approach, and be geared towards increasing peer support and community engagement.

Programs could be created for WLWH that provide information regarding the law surrounding HIV nondisclosure, while also focusing on the sharing of disclosure navigation and documentation strategies. These initiatives could be tailored to address topics commonly WLWH’s concerns and the vulnerability they experience under the law (i.e. privacy, the potential for exploitation or violent reactions from partners, HIV stigma, and impacts on family members and children). Similar programs could be created focusing on the experiences of straight men.

Similarly, these findings could inform initiatives for racialized PLWH, and encourage connection and interaction with ASOs. In addition to providing information regarding the law, these programs could include peer sharing about ways to navigate disclosure and the law, documentation techniques, and ways to contend with HIV stigma and conservative values in their families and communities. These programs would be of particular relevance for racialized newcomers living with HIV to help them learn about the law, its requirements, their responsibilities, and ways to protect themselves (via disclosure documentation) from potential deportation.

While GBMSM report more awareness regarding the law, adeptness navigating its impacts, and innovation regarding disclosure strategies, they remain impacted–particularly through the law’s validation of HIV stigma. Programming for GBMSM could focus on sharing disclosure and documentation strategies and addressing their common concerns (i.e. the potential for blackmail, false accusations, and abuse).

Fear of criminalization and HIV stigma lead many PLWH to live single, celibate lives. From both legal and medical perspectives, this precaution is not necessary. Increasing awareness of the various ways one can navigate the impacts of the law (and document disclosure) could help support all PLWH and enable them to feel more secure and less vulnerable during their pursuit of connection and partnership. This could facilitate their access to the many potential emotional, psychological, and material benefits of relationships.

HIV criminalization, as a public health strategy, is a failure. Prevention efforts would be better served by focusing on regular testing, connecting people who have acquired HIV with treatment and community support, and by combatting HIV stigma. Institutionalized by HIV criminalization, HIV stigma is a public health issue that deters testing, erodes prevention efforts, and brings anguish into the lives of PLWH.

## Conclusion

HIV criminalization in Canada fuels and validates HIV stigma and produces vulnerability both within and outside of the relationships of PLWH. Concern for criminalization is omnipresent and ubiquitous, with many PLWH fearing the implications of disclosure and criminalization, even when following the law. Through this concern, the sexual and romantic desires of PLWH become filtered through a legal lens. Despite the law complicating relationships and dating practices, PLWH have created a breadth of navigation strategies that they utilize to protect themselves from the law. While access to these navigation strategies varies along lines of gender, race, and sexual orientation, these navigation strategies themselves are forms of innovation and resilience.

## Supporting information

S1 File (DOC)
