# Supplementary material for: Life and love under criminalization: The experiences of people living with HIV in Canada
Source: PLoS One. 2024 Jul 25;19(7):e0306894. doi: 10.1371/journal.pone.0306894 (PMC11271884; doi:10.1371/journal.pone.0306894)
Supplement: S1 File — (DOC) [file pone.0306894.s001.doc]

Before we begin, do you have any questions for me or concerns about the interview?

Please remember that if you feel uncomfortable with any of these questions, we can just skip them. Also, you can stop the interview at any time and I can get rid of your answers. It’s not a problem.

What *pseudonym* or *fake name* would you like to use for the interview?

So how would you describe your sexuality? (straight/gay/bi/queer/poly/triad/etc.)

- How would you describe your gender identity? (male/female/trans/mtf/etc.)
- When did you become sexually active?
- How long have you been positive?
- Do you have any concerns about HIV?

*Knowledge of Laws*

- What is your understanding of the non-disclosure laws? How do think they work?
- Have you heard about the 2012 Supreme Court decision? If so, what do you think about it?
- Are there any potential problems that you see with the non-disclosure laws? Are there any potential benefits?
- Do you have any concerns with the law? When did it become a concern for you?
- Should non-disclosure be criminalized? How do you think it should work?
- As the non-disclosure laws seem to be here to stay for a while (as they were not struck down by the Supreme Court), how do you think they could be better?
- How do you learn about the laws? (media, news, cases, friends, ASOs, etc.)
- Why do you think the non-disclosure laws exist? Where do you think they came from?
- Do you think the non-disclosure laws have an effect on how HIV positive people?
- Do you think they have an effect on how PHAs are viewed by society?

*Disclosure*

Now, although this is not necessarily my viewpoint, non-disclosure is illegal (under certain circumstances) and so when talking about non-disclosure you should not tell me specifics like who, when and where. You can still talk about non-disclosure, but please do so generally. Also, I can easily go back and delete bits, or I can easily turn off the recorder, for a bit if you’d like.

- How does disclosure happen (or not happen)?
- What factors into the decision to disclose or not?
- When is disclosure appropriate? When is it not appropriate?
- Is it different for sexual versus romantic relationships?
- Does disclosure have anything to do with the law, in your experience?
- Do the non-disclosure laws affect you?

*Experience with Law*

- Have you, or your friends, had any experiences with the law?
- Do you feel that the law is something that you have to protect yourself from?
- If so, what steps do you take to protect yourself from the law, if any?
- Have you ever discussed the non-disclosure laws with your friends/peers? How do they deal with them?

*Section 22 Orders*

- Have you heard of a s.22 order? <if not, then read description below>
- Do you have any concerns about s.22s?
- Are there any potential problems that you see with the use of s.22s? Are there any potential benefits?
- Have you, or any of your peers, had any experiences with s.22?
- Some people take steps to avoid s.22s. Have you heard of anything like that?

*Community*

- Do you think the non-disclosure laws have had an effect on the LGBT community?
- Do you think that the non-disclosure laws have an effect upon PHAs?
- Do you think the LGBT community should respond to the non-disclosure laws?
- Have you seen any sort of response?
- In your opinion, are the non-disclosure laws affect interaction between negative and positive people?

*Citizenship & Human Rights*

- Are you a Canadian citizen?
- Is Canada a good place to live for PHAs?
- Do you feel that the non-disclosure laws infringe upon human rights?
- What do you think about how Canada treats PHA? Like, what does Canada do well, and what could it do better?
- What do you think about Canada’s treatment of human rights?
  - - *What rights do you feel that you have? Are there any rights that you feel that others have that you don’t?*
- Do you think that having HIV changes your rights?
- Do you think that having HIV changes how the government treats you?
- *Like, does it protect/respect/infringe upon/ignore your rights?*
- Do you think that Canada respects the rights of PHAs?
- Do you feel that Canada treats some people better than others?
- Do the non-disclosure laws change what you think about Canada?

*Messages & Views*

- What messages do the non-disclosure laws send?
- What do you think negative people think about the non-disclosure laws?
- What do you think positive people think about the non-disclosure laws?
- Do the non-disclosure laws affect how you see or feel about yourself?
- Do the non-disclosure laws change how you see your sexuality?
- Do the non-disclosure laws affect your approach to sex?
- Do the non-disclosure laws affect your approach to relationships?
- Do the non-disclosure laws change how you think of HIV?
- Do you think the non-disclosure laws change how other people see HIV?
- Do the non-disclosure laws have any effect on your everyday life, do you think?

*Prevention*

- What kinds of interaction have you had with AIDS Service Organizations (ASOs), if any?
- Have you used any of their services or participated in any of their programs/interventions?
- What kinds of programs? (prevention, workshops, counselling, etc.)
- What motivates your taking part (or NOT taking part)?
- Do you have any concerns about participating in ASO programs?
- What goes through your mind when thinking about signing up for an ASO program?
- Do you feel that the non-disclosure laws have any effect upon how you interact with ASOs?
- When you/if you were to participate with ASO work, do you feel that you can be honest with ASO workers about your sexual experiences?
- Do concerns about the non-disclosure laws cause you to contour what you say to ASOs, health professions, friends, peers?
- Do you think the nondisclosure law can stop some PHAs from participating in programs?
- Hypothetically speaking, if a positive person were to mention an example of non-disclosure to an ASO worker, what do you think would happen? (cops, public health)
- What do you think ASOs could do to deal with this perception?

*Demographics*

Now I’d like to talk about some demographical information. This helps me to see the variety of people who’ve participated in the study.

- Citizenship (asked above)
- Where do you live, like in what city?
- How would you describe your race or ethnicity? Use as many as you feel suit you.
- What’s your level of education? (some high school/high school/college/ university/graduate/etc.)
- How old are you approximately? (20s/30s/40s/50s/60s/etc.)
- What’s your approximate annual income? (ODSP/20-40/40-60/60-80/80-100/100+)
- How did you hear about this study?

*Closing*

We’re done! Do you have questions or comments for me about the interview or research process?

- What did you think of the interview?
- Are there any questions you think I should have asked?
- Are there other topics or issues you think we should discuss?
- Would you like to receive the findings of the study? I can email them to you.

*Section 22 Description*

- Under Ontario’s Health Protection and Promotion Act, Public Health can order a person to do or refrain from doing anything reasonably necessary to decrease or eliminate a public health risk, including ordering someone to have a physical examination, to reveal the names of sexual or drug contacts or, to refrain from activities that put others at risk – like sex. Public Health can consider a s.22 order if someone appears to putting others at risk (through continued unprotected sex, for example). Public Health will consider a s.22 order if a PHA tests positive for other STIs (which indicates unsafe sex) or if there is a complaint by a private citizen. S.22s are confidential (not public record, like criminal charges) and can be appealed in a hearing before the Health Services Appeal and Review Board.
